# Nitric oxide alters hyaluronan deposition by airway smooth muscle cells

**DOI:** 10.1371/journal.pone.0200074

**Published:** 2018-07-02

**Authors:** Alana K. Majors, Ritu Chakravarti, Lisa M. Ruple, Rachel Leahy, Dennis J. Stuehr, Mark Lauer, Serpil C. Erzurum, Allison Janocha, Mark A. Aronica

**Affiliations:** 1 Department of Pathobiology, Lerner Research Institute, Cleveland Clinic, Cleveland, Ohio, United States of America; 2 Respiratory Institute, Cleveland Clinic, Cleveland, Ohio, United States of America; 3 Lerner Research Institute, Cleveland Clinic, Cleveland, Ohio, United States of America; 4 Department of Biomedical Engineering, Lerner Research Institute, Cleveland Clinic, Cleveland, Ohio, United States of America; Albany Medical College, UNITED STATES

## Abstract

Asthma is a chronic inflammatory disease that is known to cause changes in the extracellular matrix, including changes in hyaluronan (HA) deposition. However, little is known about the factors that modulate its deposition or the potential consequences. Asthmatics with high levels of exhaled nitric oxide (NO) are characterized by greater airway reactivity and greater evidence of airway inflammation. Based on these data and our previous work we hypothesized that excessive NO promotes the pathologic production of HA by airway smooth muscle cells (SMCs). Exposure of cultured SMCs to various NO donors results in the accumulation of HA in the form of unique, cable-like structures. HA accumulates rapidly after exposure to NO and can be seen as early as one hour after NO treatment. The cable-like HA in NO-treated SMC cultures supports the binding of leukocytes. In addition, NO produced by murine macrophages (RAW cells) and airway epithelial cells also induces SMCs to produce HA cables when grown in co-culture. The modulation of HA by NO appears to be independent of soluble guanylate cyclase. Taken together, NO-induced production of leukocyte-binding HA by SMCs provides a new potential mechanism for the non-resolving airway inflammation in asthma and suggests a key role of non-immune cells in driving the chronic inflammation of the submucosa. Modulation of NO, HA and the consequent immune cell interactions may serve as potential therapeutic targets in asthma.

## Introduction

Asthma is a multifactorial, chronic inflammatory disease of the airway whose etiology is poorly understood and that affects more than 24 million Americans (see the Center for Disease Control report for 2015 that can be found at https://www.cdc.gov/asthma/most_recent_data.htm). Airway remodeling is a hallmark of asthma with smooth muscle cell (SMC) proliferation and extensive changes to the extracellular matrix, such as the excessive deposition of collagen and hyaluronan (HA)[1–3]. Hyaluronan is a linear glycosaminoglycan composed of alternating units of β1,3-D-glucuronic acid and β1,4-N-acetyl-D-glucosamine and it has several functions relevant to inflammation[4–7]. Lymphocytes accumulate in the airways of asthmatics and have many pathological functions[8,9]. The interactions of lymphocytes with HA may contribute to their retention, activation, functional properties and survival[10,11]. Identification of mediators that contribute to the deposition of HA, as well as factors that can prevent its abnormal accumulation, may lead to the development of novel therapies for patients with asthma, particularly those who are refractory to the current treatment methods.

Hyaluronan is present in both pulmonary secretions and in the submucosa of patients with asthma[12,13]. Hyaluronan synthase-2 (HAS-2) is a major enzyme responsible for HA synthesis and has recently been shown to be a susceptibility gene for asthma[14]. Overexpression of HAS-2 in SMCs and myofibroblasts alters remodeling and hyper-reactivity in allergen-challenged mice[15]. Historically, the accumulation of HA has been viewed as simply a marker of inflammation, but the accumulation of HA may have many pathological consequences, including alteration of the biomechanical properties of the tissue, increasing the proliferation of SMCs[16], enhancing the production of transforming growth factor-beta (TGF-β)[17], modulating the effects of TGF-β[18,19], increasing the activation of tissue kallikrein[20,21], and modulation of leukocyte activity[4,22,23]. Circulating and sputum HA levels are potential biomarkers of asthma control[24], consistent with a role of HA in the pathogenesis of asthma[24,25].

Airway SMCs play a key role in the pathogenesis of asthma and are a source of HA[26]. Numerous growth factors and cytokines have been identified that induce HA synthesis such as interleukin-1β, platelet derived growth factor, tumor necrosis factor-α, fibroblast growth factor-2, and TGF-β[27]. TGF-β was recently shown to induce HA cables in lung myofibroblasts[18] and numerous reports have confirmed a role of TGF-β in the pathogenesis of asthma[28]. However, only agents that cause endoplasmic reticulum stress, double stranded RNA, and dextran sulfate promote the deposition of HA into cable-like structures by cultured SMCs[29–31]. These cable-like structures of HA, but not other forms of HA produced by SMCs, bind immune cells[29–31]. Interestingly, endoplasmic reticulum stress and double stranded RNA are associated with asthma[32–36]. The asthma-associated gene *ORMDL3* regulates endoplasmic reticulum stress[37,38], and respiratory viruses are closely associated with both development and exacerbations of asthma[39,40].

Nitric oxide is produced at high levels by the airway ciliated epithelium in asthmatics and is associated with greater airway reactivity and greater evidence of airway inflammation[41–44]. NO plays an important role in the pathogenesis of asthma and also serves as a biomarker of airway inflammation. NO has many important physiological functions, but at high levels can cause pathology[41,45]. The effects of NO are dependent on the target cell, environment and concentration of NO[46,47]. NO has clear effects on vascular smooth muscle cells including vasorelaxation and modulation of proliferation and matrix production[48], but the effects of NO on airway SMCs have not been elucidated.

Many, but not all, of the effects of NO are mediated by soluble guanylate cyclase (sGC)[49,50]. The binding of NO activates sGC, resulting in the generation of cGMP from GTP. The cGMP that is formed activates protein kinase G, which, through modulation of calcium levels, causes smooth muscle relaxation[49]. This results not only in vasodilation, but also bronchial dilation[51,52]. Numerous other effects of NO are mediated by sGC-independent mechanisms such as S-nitrosylation of thiols, nitration of tyrosine residues and nitrosylation of certain transition metals[53]. 8-nitro-cGMP has also been demonstrated to mediate some of the effects of NO via protein S-guanylation[54–56].

In the present study we hypothesized that NO leads to the production of leukocyte adhesive-HA by airway SMCs. To test this, SMCs were exposed to NO produced by a variety of sources and HA production and function were evaluated. The potential role of sGC in modulating HA deposition was examined.

## Materials and methods

### Reagents

All materials were purchased from Sigma-Aldrich (St. Louis, MO) unless stated otherwise. The tunicamycin was from *Streptomyces sp*. The NO donors S-Nitroso-N-acetyl-D, L-penicillamine (SNAP), DETA NONOate (NOC-18), NOC-12 and DEA NONOate (NOC-2) were purchased from Enzo Life Sciences International (Plymouth Meeting, PA). Murine interferon-gamma was purchased from Peprotech (Rocky Hill, NJ); and IL-1β and TNF-α from R&D (Minneapolis, MN). Sildenafil was purchased from Pfizer (New York, NY).

### Animals

This study was carried out in accordance with the recommendations in the Guide for the Care and Use of Laboratory Animals of the National Institutes of Health. All mice were maintained at the Cleveland Clinic Lerner Research Institute, Biological Resource Unit in a temperature controlled facility with an automatic light-dark cycle and were given free access to food and water. All animal protocols were approved (number 2013–1097) by the Institutional Animal Care and Use Committee (IACUC) of the Cleveland Clinic (fully accredited by the Association for Assessment and Accreditation of Laboratory Animal Care International). Mice were sacrificed by inhalation of isoflurane (Baxter, Deerfield, MI) followed by cervical dislocation. Wild type Balb/c, C57BL/6 and *iNos* KO mice (B6.129P2-Nos*2*^*tm1Lau*^/J) were purchased from Jackson Labs (Bar Harbor, ME). *Has 1*,*3* double knockout mice were the generous gift of Joe Hollyfield and Edward Maytin (Cleveland Clinic). Briefly, *Has1* knockout[57] and *Has3* knockout mice[58] were bred to generate *Has1*,*3* double knockout mice. C57 BL/6 mice with floxed *Has2* genes[59] (a generous gift from Yu Yamaguchi at Burnham Institute for Medical Research and Edward Maytin at the Cleveland Clinic) were bred with BL/6 mice containing a beta-actin-driven, Cre recombinase (B6.Cg-Tg(CAG-cre/Esr1*)5Amc/J from Jackson Labs) to generate conditional *Has2* knockout mice responsive to tamoxifen.

### Cell isolation and culture

Murine airway SMCs and epithelial cells were obtained enzymatically from excised tracheas from wild type Balb/c, wild type C57Bl/6, *iNos* knockout Bl/6, and *Has 1*,*3* double knockout mice. Briefly, the tracheas were carefully cleaned of adherent tissue under a dissecting microscope and opened longitudinally. Epithelial cells were removed by digestion overnight at 4°C with 0.15% pronase (Roche, Indianapolis, IN) in HAM’s F-12 medium (Gibco, Carlsbad, CA) with antibiotic/antimycotic (Gibco). The isolated epithelial cells were grown on an air-liquid interface as previously described[60].

The remaining tissue containing the SMCs was washed carefully with Dulbecco's modified Eagle’s/Ham's F12 (DMEM/F12) medium containing an antiobiotic/antimycotic, cut into small (<2 mm) pieces and digested in serum-free DMEM/F12 containing 0.1% type 4 collagenase from *Clostridium histolyticum* (Worthington Biochemical Corporation, Lakewood, NJ), 0.05% porcine pancreatic elastase (Worthington Biochemical Corporation) and an antibiotic/antimycotic for up to 6 hours at 37°C. Following digestion, the cells were centrifuged, resuspended and grown in DMEM/F12 medium with HEPES, 10% fetal bovine serum (FBS) (Bio-Whitaker, Walkersville, MD), and an antibiotic/antimycotic mixture (complete medium). All cultures were maintained at 37°C in 95% air and 5% CO_2_ and split at a maximal ratio of 1:3. Passages 2–3 were employed in these studies. SMCs from the *HAS2* conditional knockout mice were treated with 0–2 micromolar hydroxy-tamoxifen every other day for ten days and were then rested for two days before being plated for experiments.

### Co-culture experiments

RAW-SMC co-cultures: RAW cells (RAW 264.7 from American Type Culture Collection, Manassas, VA) were cultured on trans-wells in DMEM medium with 10% FBS and antibiotic/antimycotic until approximately 60–70% confluent and were treated with interferon gamma and lipopolysaccharide (LPS) for 6–8 h. The trans-wells were then gently washed with serum-free medium and transferred to plates containing coverslips with confluent SMCs. The co-cultures were maintained for 13 h and then the SMCs were rinsed with PBS, fixed in cold methanol for 5 min, air-dried and stained for HA. For the epithelial- SMC co-cultures, confluent epithelial cells on an air-liquid interface were treated for 24 h with 10 ng/ml IL-1β, 10 ng/ml TNF-α and 10 ng/ml IFN-γ to induce iNOS expression[61]. The inserts with the epithelial cells were carefully rinsed with serum-free medium and transferred to plates containing a coverslip with a confluent layer of wild type SMC. The co-cultures were maintained for 23 h followed by rinsing, fixing and staining of the SMCs as described for the RAW-SMCs cocultures.

### Leukocyte adhesion

SMCs were grown to confluence in 24 well plates and then treated with the appropriate test compounds for 23 h prior to the assay unless stated otherwise. The quantification of leukocyte adhesion to SMCs was performed with fluorescently labeled leukocytes. Freshly isolated murine leukocytes were obtained from spleens. Briefly, the tissue was removed, placed in RPMI 1640 medium, gently homogenized and strained through a 40 μM mesh. The red blood cells were lysed with lysis buffer (Gibco) and the leukocytes were quickly centrifuged, resuspended and washed. To deplete the samples of monocytes and macrophages, the cells were incubated for 45 min at 37°C in RPMI medium containing 10% FBS and antibiotic/antimycotic to allow those cells to attach to the plastic. The nonadherent leukocytes were resuspended in complete DMEM/F12 medium at 20 x 10^6^ cells/ml and were fluorescently labeled with 1 μM calcein AM (Molecular Probes, Eugene, OR) for 15 min at 37°C. The labeled cells were then washed extensively with the complete medium and resuspended at a concentration of 8 x 10^6^ cells/ml in cold complete medium. The leukocytes were added to the SMCs (4 x 10^6^/well) and incubated at 4°C for 30 min. Nonadherent leukocytes were removed by washing the wells with cold medium containing 3% FBS. To quantify the adhesion, cells were lysed by freeze/thawing and the fluorescence was measured in a fluorimeter. In a typical experiment, 10–40% of the added leukocytes adhered to treated cultures. Some cultures were treated with 200 μg/ml bovine testicular hyaluronidase type IV-S for 3–5 minutes prior to the final wash to quantify the number of cells bound to HA.

### Leukocyte binding to SMC cultures

Murine leukocytes were obtained as described above and depleted of monocyte/macrophages by allowing those cells to attach to plastic. The nonadherent cells were resuspended in cold DMEM/F12 medium with 10% FBS. The leukocytes were incubated with SMC cultures for 30 min at 4°C. The nonadherent leukocytes were removed and the SMCs with adherent leukocytes were rinsed in PBS, fixed in cold methanol and air dried. For experiments involving fixed leukocytes, the leukocytes were isolated and fixed for 10 min in 10% buffered formalin, rinsed in cold medium with 10% FBS and exposed to SMCs for 30 min at 4°C. The cultures were rinsed to remove nonadherent leukocytes, fixed in 10% buffered formalin and then rinsed with PBS. The coverslips were mounted on slides and examined by phase contrast microscopy with a Leica microscope.

### Affinity histochemistry

SMCs were grown on glass coverslips and, at the time of harvest, were fixed with -20°C methanol and air-dried. Specimens were rehydrated in PBS and nonspecific binding was blocked with PBS containing 3% FBS. Samples were then incubated with 5 μg/ml biotinylated HA-binding protein (EMD Millipore, Billerica, MA) in PBS containing 3% FBS. After extensive washing with PBS containing 3% FBS, 4 μg/ml Alexa 488-conjugated avidin (Molecular Probes) was added. The samples were then extensively washed and mounted with mounting medium containing 4’,6 diamidino-2-phenylindole (Vector Laboratories, Burlingame, CA) to counterstain the nuclei. Samples were examined with a confocal microscope equipped for fluorescence. The HA-binding protein was omitted in assays serving as negative controls. Images were acquired using a Leica TCS-SP2 scanning confocal microscope equipped with four lasers, three PMT detectors and a spectrophotometer, operated with Leica Confocal Software and using an HC PL APO 20x/0.7 objective and an HCX PL APO 40x/1.25 objective (Leica Microsystems, GmbH, Wetzlar, Germany).

### NO analysis

Concentrations of nitrite and nitrate in conditioned medium were measured in duplicate by a dedicated HPLC system (ENO-20; Eicom, San Diego, CA)[62]. Nitrite and nitrate concentrations were calculated based on authentic standards. The sum of nitrite and nitrate concentrations was used to reflect total NO levels.

### HA solid phase binding assay

HA was quantified as described[63] with slight modifications. Briefly, Immulon II high protein binding, 96-well plates were coated overnight at room temperature with 5 μg HA in 20 mM sodium carbonate, pH 9.6. Wells were washed in PBS/Tween and blocked with 1% BSA for at least one hour at room temperature. After washing, the experimental samples were added in a volume of 100 μl, followed by the addition of 100 μl biotinylated-HABP (1 μg/ml in 100 μl PBS/Tween). The plates were incubated for at least 90 min at room temperature and then washed with PBS/Tween. Horseradish peroxidase-conjugated streptavidin (Thermo-Fisher, Rockford, IL) was added for at least 30 min and the wells were washed, followed by incubation with 200 μl *o*-phenylendiamine (SigmaFast OPD) until substantial color developed (approximately 5 min). The reaction was stopped by the addition of 50 μl 3N HCl. Absorbance was read at 492 nm and sample concentrations were calculated from a standard curve containing 10 ng/ml– 10 μg/ml rooster comb HA. Each sample was run in duplicate.

### Western blot analysis

Electrophoresis was performed with 4–20% mini-protean TGX gels (Bio-Rad, Hercules, CA) and proteins were transferred to PVDF membranes with the Bio-Rad Trans-Blot Turbo Transfer system. Blots were blocked with Tris-buffered saline containing 1% Tween-20 (TBS-T) and 3% bovine serum albumin for an hour at room temperature. Blots were incubated with anti-HAS2 antibody (Aviva Biosystems) overnight at 4°C or with anti-GAPDH (Cell Signaling Technology, Danvers, MA) for one hour at room temperature. Three fifteen minutes washes in TBS-T were performed before applying a goat anti-rabbit antibody conjugated to horseradish peroxidase (Cell Signaling Technology) for one hour at room temperature. The blots were then washed three times for fifteen minutes per wash in TBS-T and chemiluminescence was detected with the Clarity system (Bio-Rad) and a Gel-doc imaging system (Bio-Rad) or a ThermoFisher imaging system. After detecting HAS2, the blots were stripped with One Minute Stripping Buffer (GM Biosciences, Rockville, MD), rinsed for 15 minutes in TBS-T, and blotted for GAPDH.

### Statistics

*P* values were calculated with Quattro Pro for Windows using a two-tailed Student’s T test. Differences were considered significant at *P* < 0.05.

## Results

**Fig 1:**
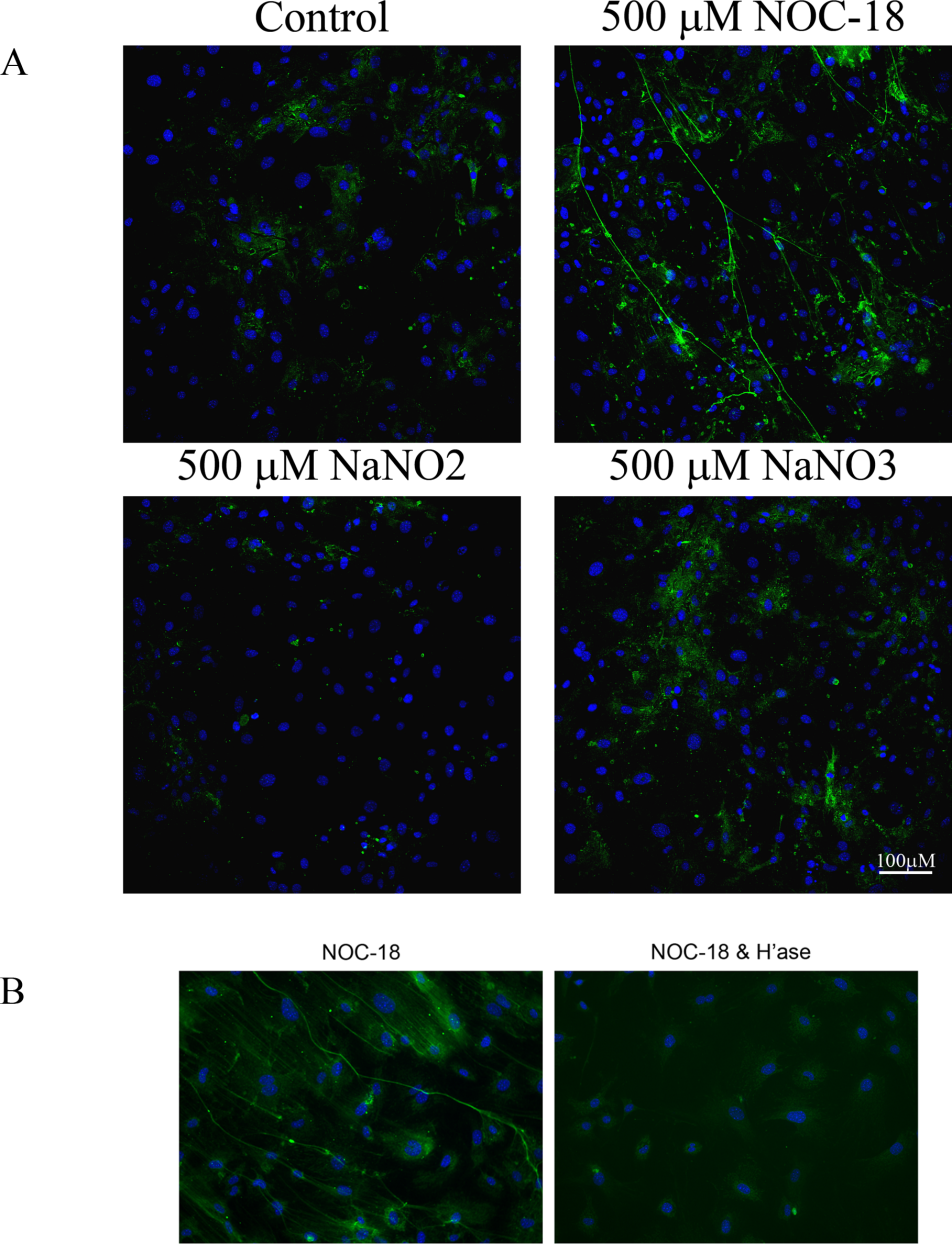
NOC-18 promotes the deposition of HA cables. (A) Wild type Balb/c airway SMCs were treated for 23 h at 37°C with or without 500 μM NOC-18, NaNO_2_ or NaNO_3_. The cell layer was rinsed in PBS, fixed in cold methanol and stained for HA (green) and nuclei (blue). Original magnification– 20X. (B) Wild type Bl/6 airway SMCs were treated for 23 h at 37°C with 500 μM NOC-18 and then without or with 200 μg/ml bovine testicular hyaluronidase type IV-S for 3–5 minutes. The cell layer was rinsed in PBS, fixed in cold methanol and stained for HA (green) and nuclei (blue). Original magnification– 20X. Images shown are representative of experiments with at least three separate isolates of SMCs.

To determine if NO affects HA deposition, murine airway SMCs from wild type Balb/c mice were treated for 23 hr with the NO donor NOC-18 (500 μM) and were then stained for HA. NOC-18 has a half-life of approximately 20h under the culture conditions and mediates a slow release of NO during the culture period[64]. 500 μM NaNO_2_ or 500 μM NaNO_3_ were used as controls. The HA from untreated, control SMCs is found to be primarily cell-associated with no obvious structure (Fig 1). In contrast, NOC-18 results in the deposition of HA in the form of long, cable-like structures (Fig 1). Hyaluronan in SMC cultures treated with 500 μM NaNO_2_ or 500 μM NaNO_3_ is comparable to that in untreated controls. Airway SMCs from wild type C57BL/6 mice also produce HA cables in response to NOC-18. The cables are no longer detected after treatment with hyaluronidase.

**Fig 2:**
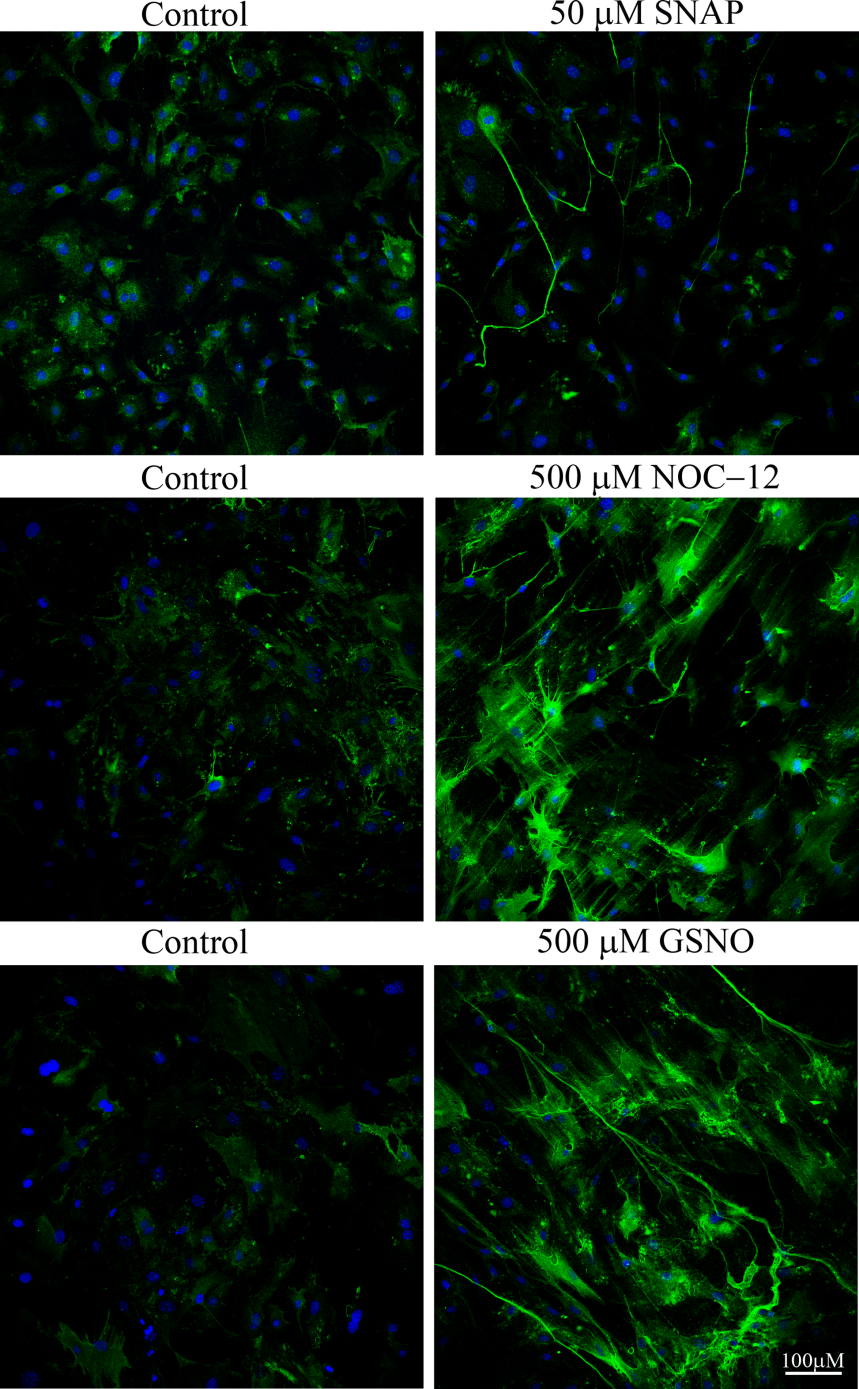
NO promotes the deposition of HA cables. SMCs were treated for 23 h at 37°C with or without 50 μM SNAP, 500 μM NOC-12, or 500 μM GSNO. The cell layer was rinsed in PBS, fixed in cold methanol and stained for HA (green) and nuclei (blue). Original magnification– 20X. Images shown are representative of experiments with three separate isolates of SMCs.

We next examined the effects of a quick, transient dose of NO on HA deposition by employing SNAP, which has a half-life of only a few minutes under the experimental culture conditions (37°C, neutral pH). Smooth muscle cells treated with a single dose of SNAP (50 μM) produce HA cables, demonstrating that even short bursts of NO exposure are adequate to induce HA deposition by SMCs (Fig 2). Treatment of SMCs with NOC-2 (half-life = 2 min) produces HA cables as well (not shown). NOC-12, which has a half-life of 100 min, also promotes deposition of HA cables (Fig 2), as does GSNO, a molecule that transfers NO rather than releasing it directly into the medium (Fig 2). NOC-18 results in a steady, slow release of NO over a long time period, so this NO donor was employed for the remainder of our studies.

**Fig 3:**
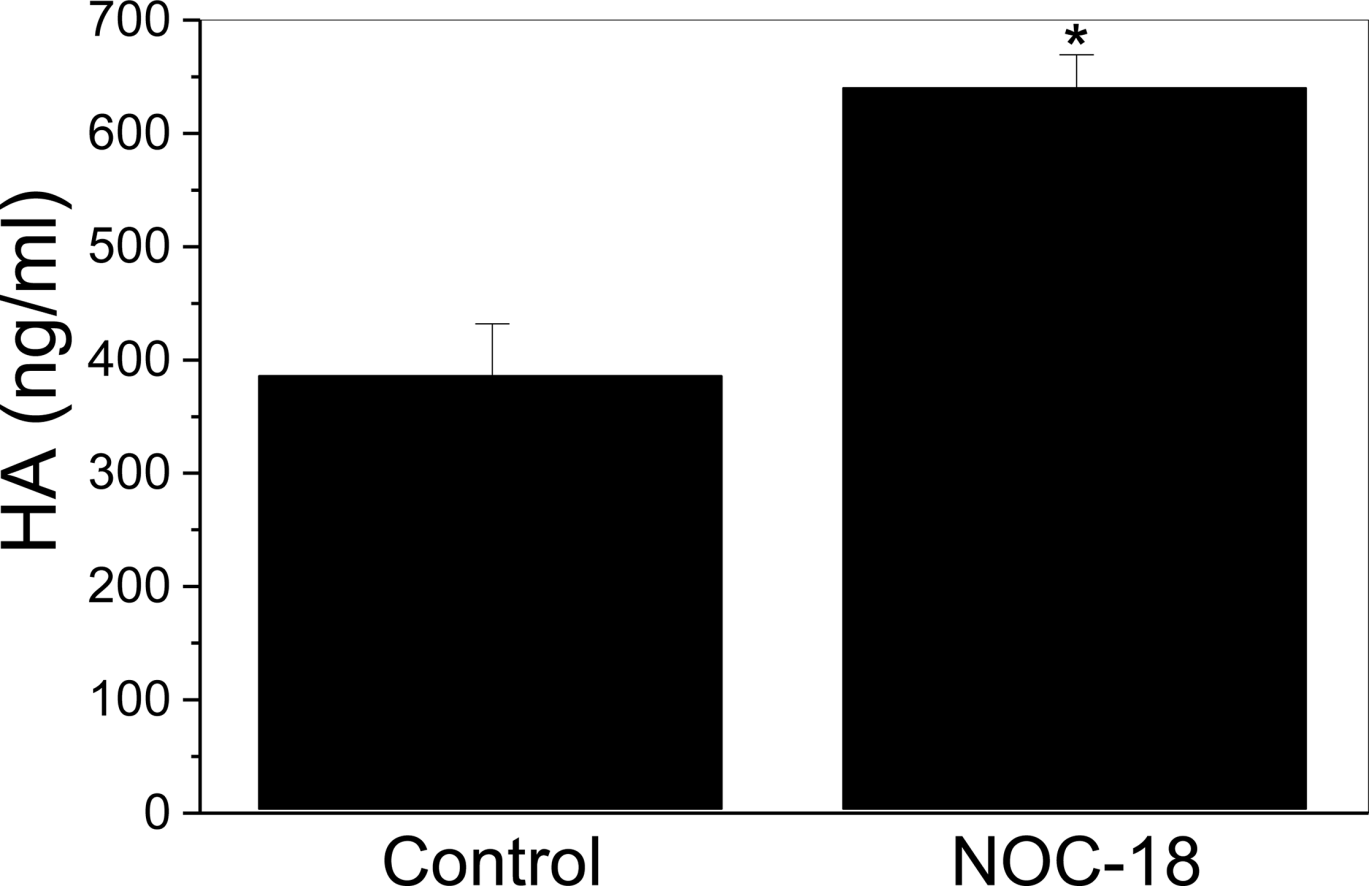
NO increases HA production. SMCs were treated with 500 μM NOC-18 for 6 h. Conditioned medium was collected and HA was quantified with a solid phase binding assay. Data represent the mean +/- S.D. n = 6, *P* < 0.0001.

To determine if HA is increased by NO, or if the HA is simply deposited in a different structural form, HA was quantified by a solid phase binding assay. Fig 3 shows that HA increases with increasing time, suggesting that new HA synthesis is occurring. SMC cultures treated with NOC-18 for 6 hours contain significantly more HA than control cultures (p<0.0001).

**Fig 4:**
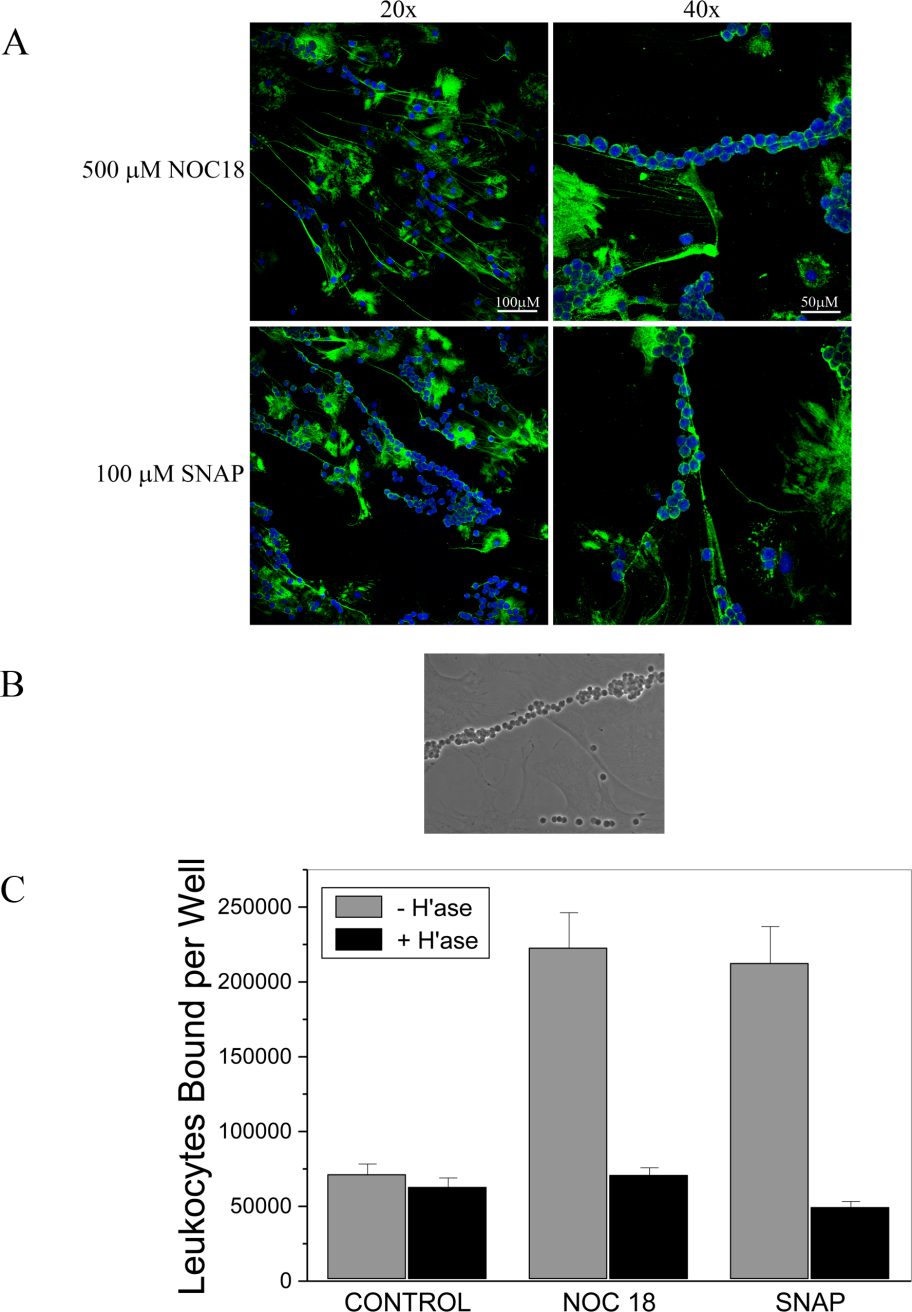
NO promotes HA-mediated leukocyte binding. SMCs were treated with 500 μM NOC-18 or 100 μM SNAP for 23 h. The cultures were rinsed with PBS and unlabeled, fixed, or fluorescently labeled murine splenocytes were added for 30 min at 4°C and nonadherent cells were removed. (A) The SMC layer with unlabeled, adherent leukocytes was fixed, and stained for HA (green) and nuclei (blue). Original magnification 20x (left) or 40x (right). Images shown are representative of experiments with at least three separate isolates of SMCs. (B) The SMC layer with formalin-fixed, adherent leukocytes was fixed in formalin, rinsed, the coverslip was mounted onto a slide and examined by phase contrast microscopy. Original magnification 40x. The image shown is representative of experiments with two isolates of SMCs and two isolates of leukocytes. For each experiment, SMCs on three coverslips were used. (C) The SMC layer with adherent fluorescent leukocytes was freeze-thawed and the bound leukocytes were quantified. In some wells, the cell layer was treated with hyaluronidase after the attachment of splenocytes, and released leukocytes were removed by rinsing. Data represent the mean +/- S.D. n = 6, *P* < 0.05.

The HA cable-like structures produced in response to NO are similar to those produced by SMCs treated with tunicamycin[29,60], a potent inducer of ER stress, or with poly (I:C), a double stranded RNA mimetic[30,31]. Our previous studies have demonstrated that the HA cables, as opposed to other structural forms of HA, bind leukocytes, specifically U937 monocytic-like cells[29,60]. Staining demonstrates that HA cables produced in response to NOC-18 or SNAP bind murine leukocytes that have been enriched for lymphocytes (Fig 4A), consistent with previous studies on HA cables and monocytes[29,30]. Fixed leukocytes also bind to the HA cables (Fig 4B). The binding of primary immune cells to the NO-treated SMC cultures was quantified to confirm the binding is HA-dependent. After stimulating the production of HA cables, the SMC cultures were incubated with fluorescently labeled immune cells. Following binding and removal of unbound leukocytes, half the wells were treated with hyaluronidase to remove HA-bound leukocytes. NOC-18 treatment induces leukocyte adhesion (Fig 4C). The majority of bound leukocytes are removed with hyaluronidase treatment (Fig 4C) demonstrating that HA-containing structures mediate the attachment.

**Fig 5:**
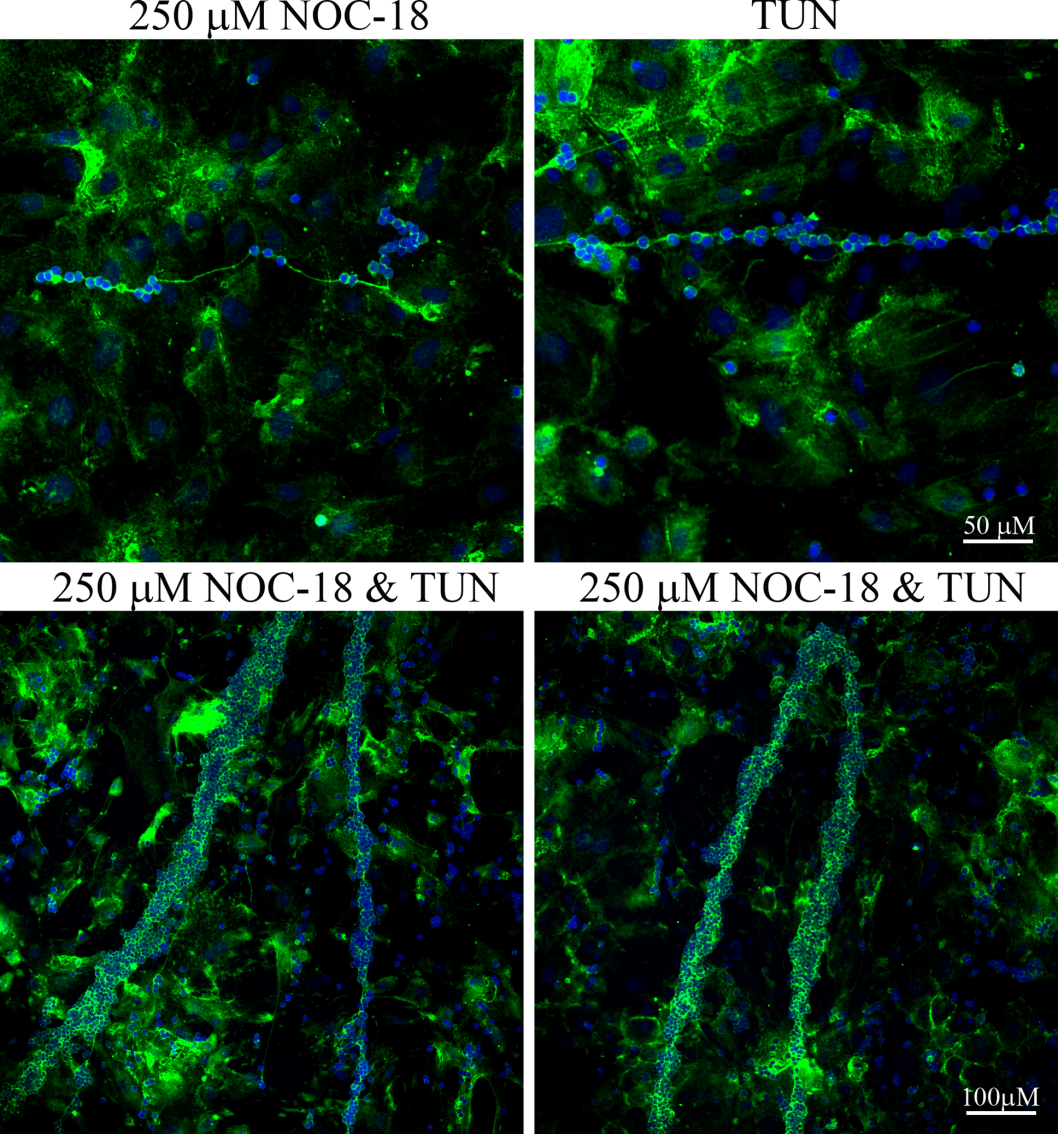
Effect of NO and ER stress on leukocyte adhesion. SMCs were treated with or without 500 μM NOC-18, 5 μg/ml tunicamycin, or both, for 23 hours. The cultures were rinsed and lymphocytes were added for 30 min at 4°C. Nonadherent cells were removed and the cultures were stained for HA (green) and nuclei (blue). Original magnification– 20X. Images shown are representative of three experiments.

*In vivo*, NO does not act alone, but in combination with other stimuli, so we treated SMCs with NO and tunicamycin, alone, and in combination, to better reflect the asthmatic SMC environment (high NO and ER stress). The SMCs treated with both NOC-18 and tunicamycin had even more leukocyte binding to HA cables than with either agent alone (Fig 5). These data suggest that *in vivo*, multiple factors associated with asthma may contribute to the alteration of HA production and the ultimate functional properties of the deposited HA.

**Fig 6:**
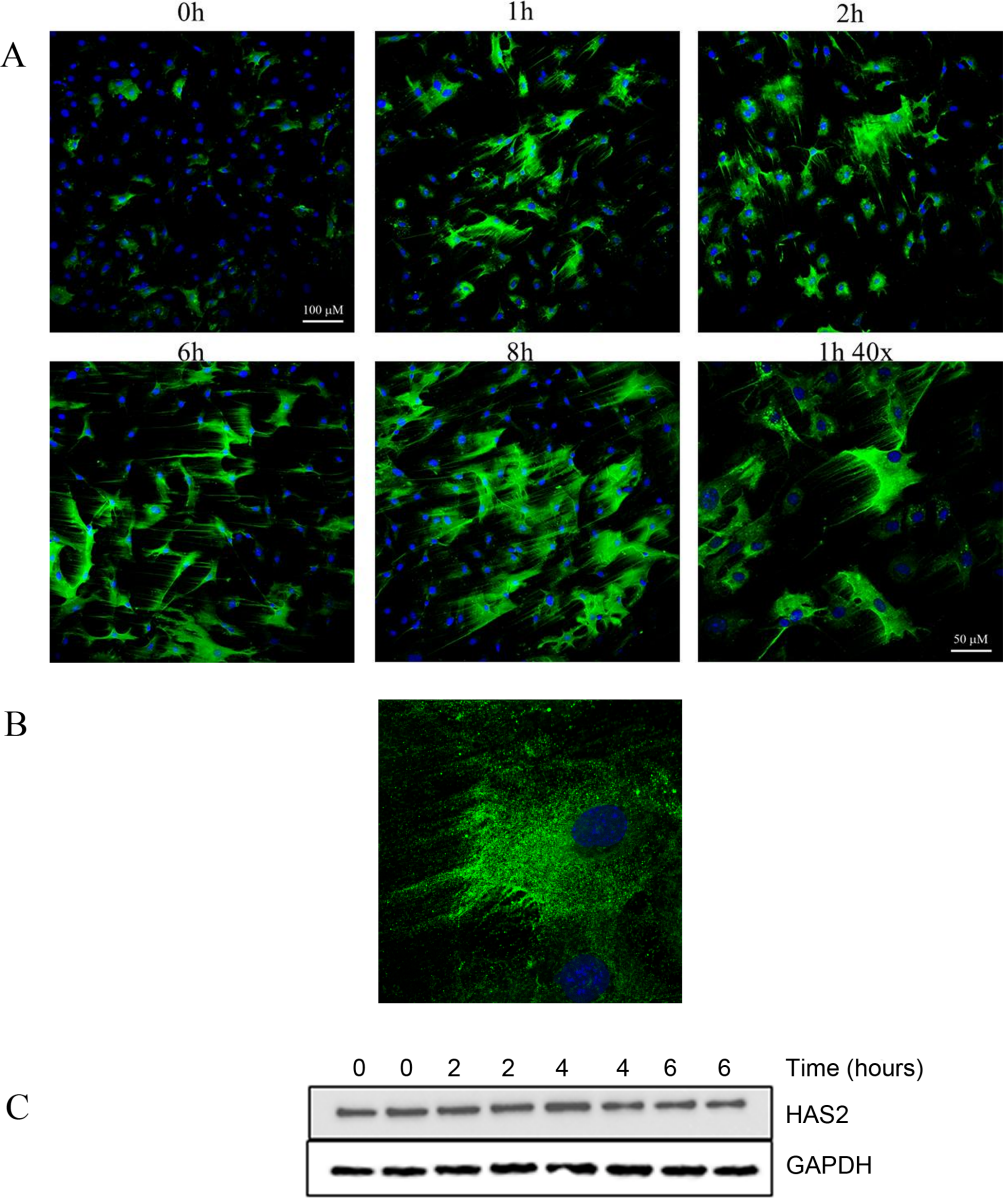
The effect of NO is rapid. (A, B) SMCs were treated with 500 μM NOC-18 for up to 8 h. The cell layers were rinsed and stained for HA (green) and nuclei (blue). Arrows point to HA-containing cables. Original magnification 20x, 40x (A), or 63x with a 2x zoom (B). Images shown are representative of experiments performed at least three times. (C) SMCs were treated with 500 μM NOC-18 for up to 6 h. Cell layers were rinsed in PBS, lysed and analyzed by Western blotting for HAS2 and GAPDH. Blots shown are representative of three experiments.

**Fig 7:**
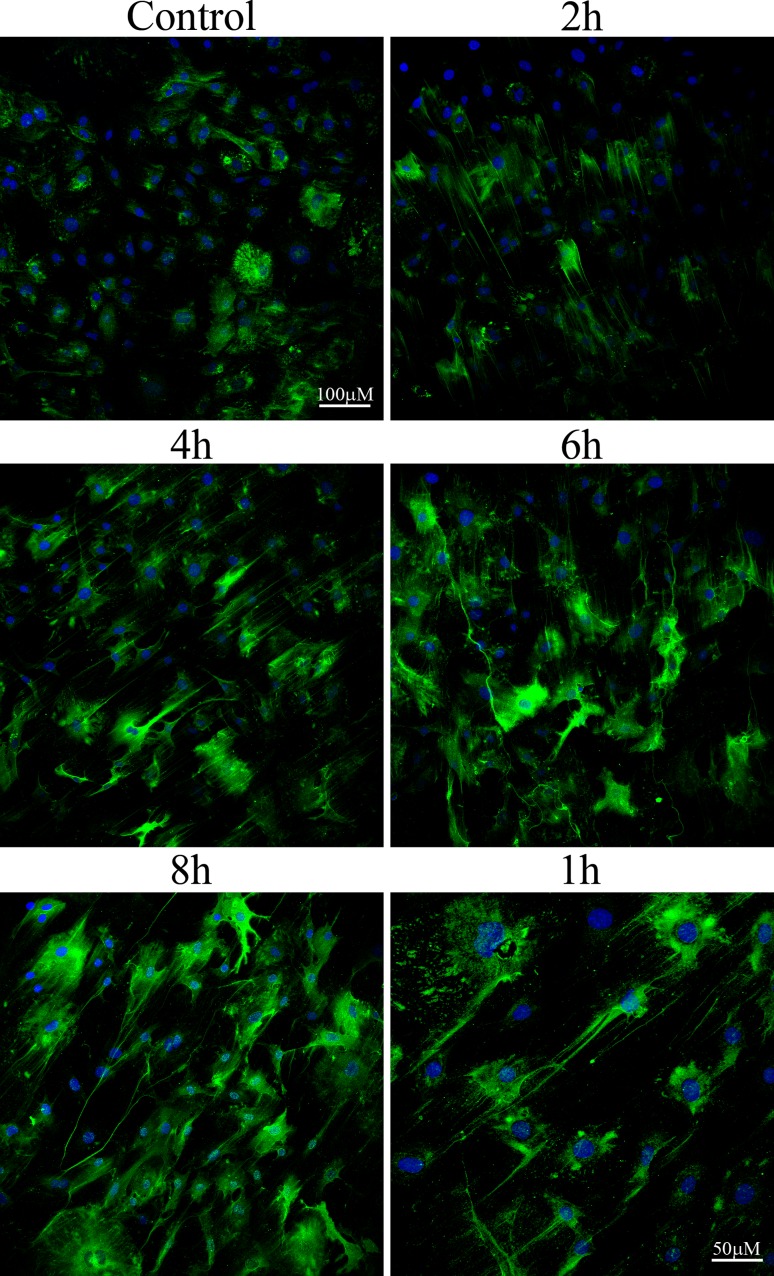
HAS 2 is sufficient for HA cable production in response to NO. *HAS 1*,*3* double knockout SMCs were treated with 500 μM NOC-18 for various times. The cultures were rinsed, fixed in cold methanol, air dried and stained for HA (green) and nuclei (blue). Original magnifications –20x. Images shown represent more than three independent experiments.

To determine how rapidly HA synthesis occurs we performed a time course analysis. HA deposition in response to NO occurs rapidly as shown in Fig 6A, where SMCs treated for time periods as short as 1 h deposited clear HA cables. Even after only 30 min projections consistent with emerging cables were present (Fig 6B). These results suggest that HA cable production in response to NO may not require the de novo synthesis of hyaluronan synthase (HAS) enzymes and may simply involve the activation of existing enzymes. Western blot analysis demonstrated that HAS2 protein levels were unchanged during the first 6 h of treatment with NOC-18 (Fig 6C). Airway SMCs obtained from *HAS1*,*3* double knock-out mice also rapidly produce HA cables in response to NOC-18 (Fig 7) demonstrating that HAS2 is sufficient to mediate the effects of NO on HA deposition.

**Fig 8:**
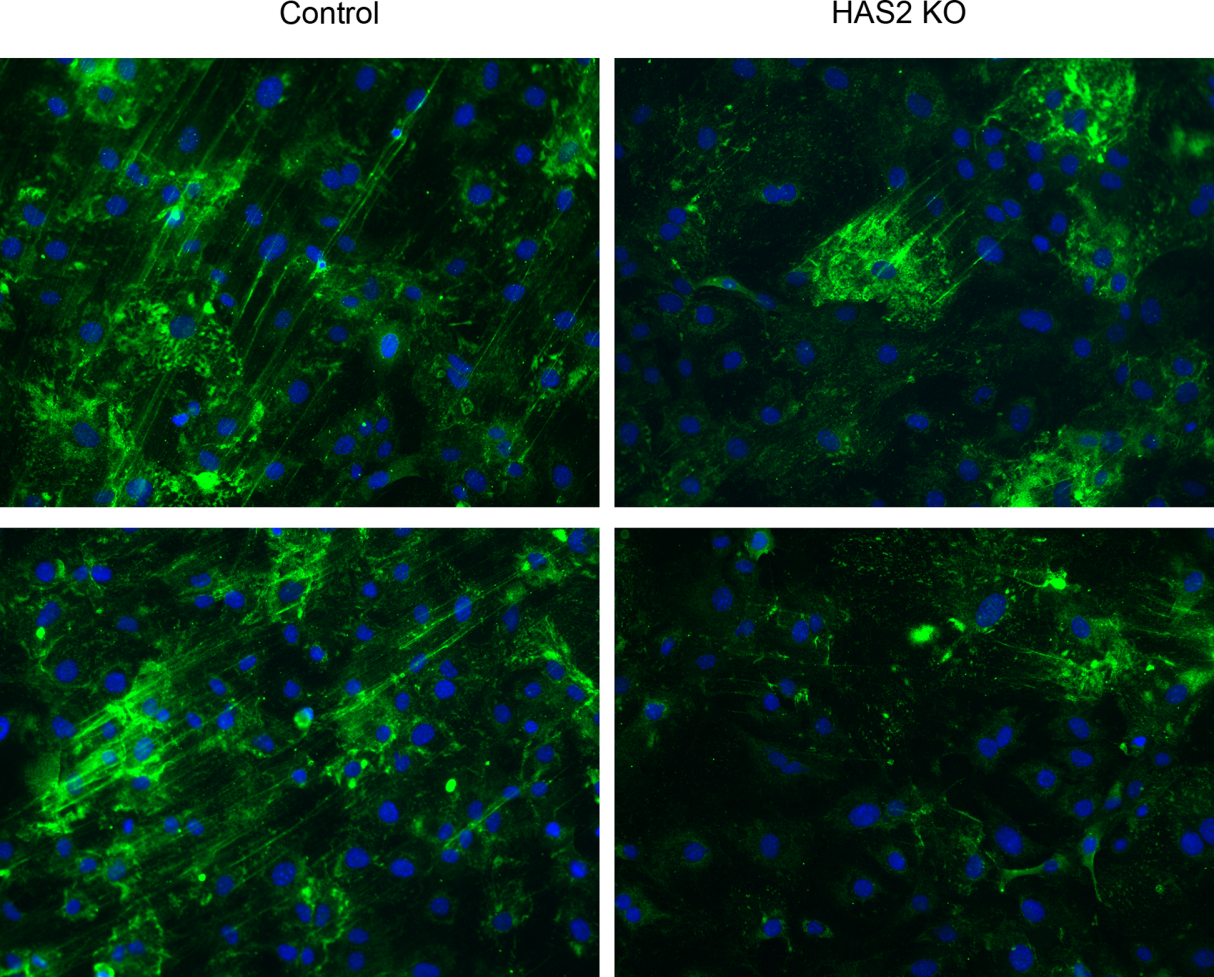
Reduced HAS2 expression limits HA cable formation. SMCs from HAS2 conditional knockout mice were cultured without or with 2 micromolar hydroxy-tamoxifen for 10 days. The cells were rested for two days and then plated on coverslips. Cultures were treated with 500 μM NOC-18 for 23 h. The cultures were rinsed, fixed in cold methanol, air dried and stained for HA (green) and nuclei (blue). Original magnifications –20x. Images shown represent experiments from three different isolates of SMCs.

To examine the contribution of HAS2 to HA cable production, airway SMCs from HAS2 conditional knockout mice were employed. The SMCs were cultured without and with 2 micromolar hydroxy-tamoxifen for ten days and were then rested for two days and passaged. Hyaluronan secretion into the conditioned medium was quantified and found to be 408 ng/ml in the untreated cultures and 152 ng/ml in the cultures treated with hydroxy-tamoxifen to reduce HAS2 expression. NOC-18 treatment of the control cultures resulted in the production of numerous HA cables (Fig 8). In contrast, the tamoxifen-treated cultures deficient in HAS2 had dramatically reduced HA cables that appeared to originate from just a few cells that likely still expressed HAS2 (Fig 8). This suggests that HAS2 is the primary HAS mediating cable formation in this *in vitro* system.

**Fig 9:**
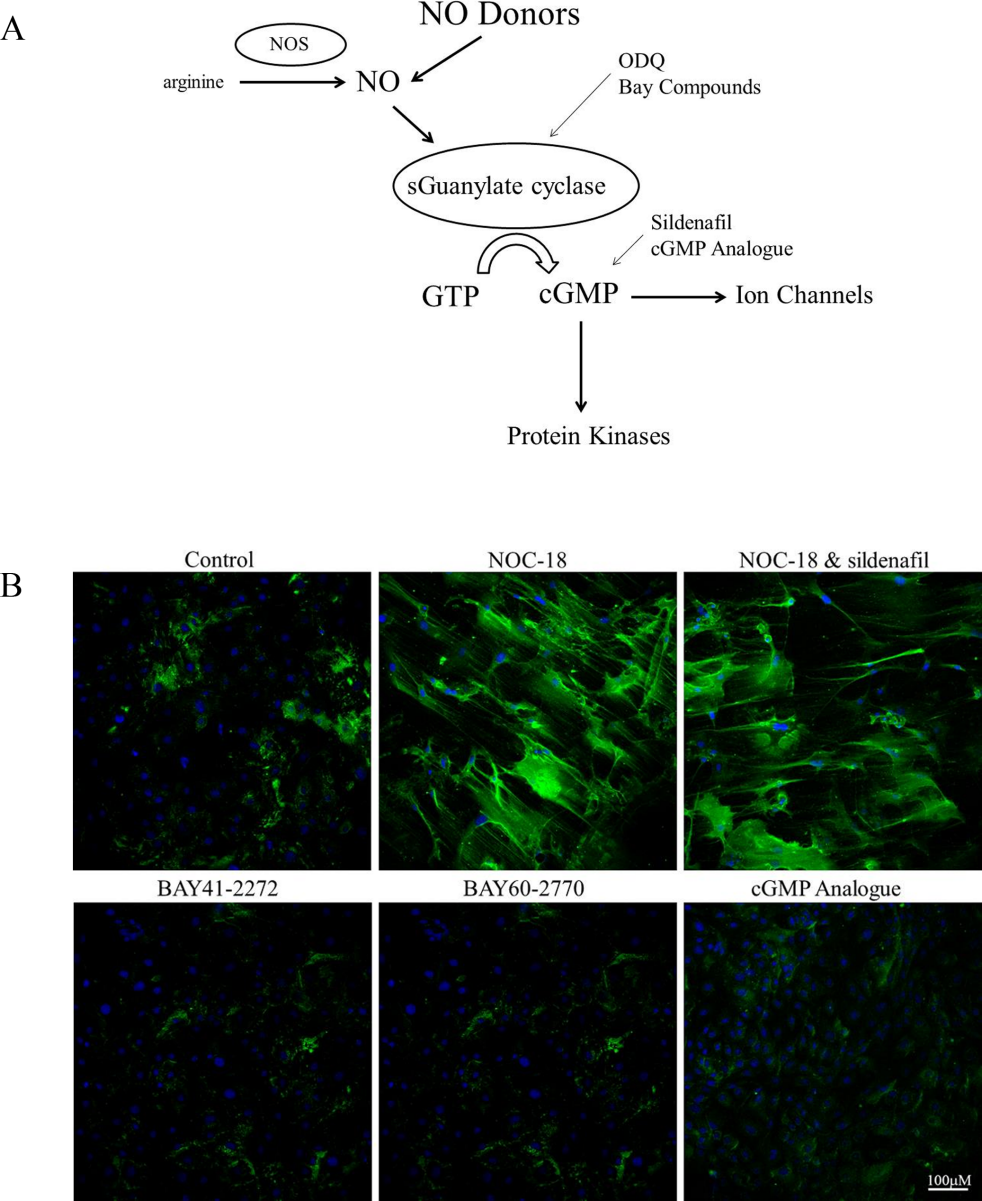
sGC does not mediate the effects of NO on HA. A) A model showing how sGC mediates the effects of NO in SMCs and compounds that can modify or mimic the effects. B) SMCs were treated with 300 μM NOC-18 alone for 6 h; pretreated with 5 μM sildenafil for 30 min and then with 5 μM sildenafil and 300 μM NOC-18 for 6 h, 20 μM BAY 41–2272 or BAY 60–2770 for 6 h; or 250 μg/ml 8-bromoguanosine 3’5’cyclic monophosphate for 6 h. At the end of the incubation the cell layers were rinsed with PBS and fixed in cold methanol, and stained for HA (green) and nuclei (blue). Original magnification - 20x. Images shown represent three separate experiments.

Although NO is known to signal through multiple pathways, the primary mediator of NO is soluble guanylate cyclase (sGC) (Fig 9A). To determine if the effects of NO on HA deposition are mediated by sGC several approaches were taken. BAY compounds are novel compounds that have been shown to directly activate sGC. SMCs were treated with 20 μM BAY 41–2272 or 20 μM BAY 60–2770 to directly activate sGC, but HA deposition was not affected (Fig 9B). Pretreatment of SMCs with 0.5 μM ODQ, a potent, irreversible inhibitor of sGC, also did not prevent the accumulation of HA cables by NOC-18 (not shown). SMCs treated with both NOC-18 and 5 μM Sildenafil to inhibit phosphodiesterase 5 (and stabilize cGMP levels) did not contain more HA cables than cultures treated with NOC-18 alone (Fig 9B). Finally, directly adding a stable analogue of cGMP had no effect on HA deposition by SMCs (Fig 9B). Together these results indicate that sGC does not mediate the effects of NO on HA deposition.

**Fig 10:**
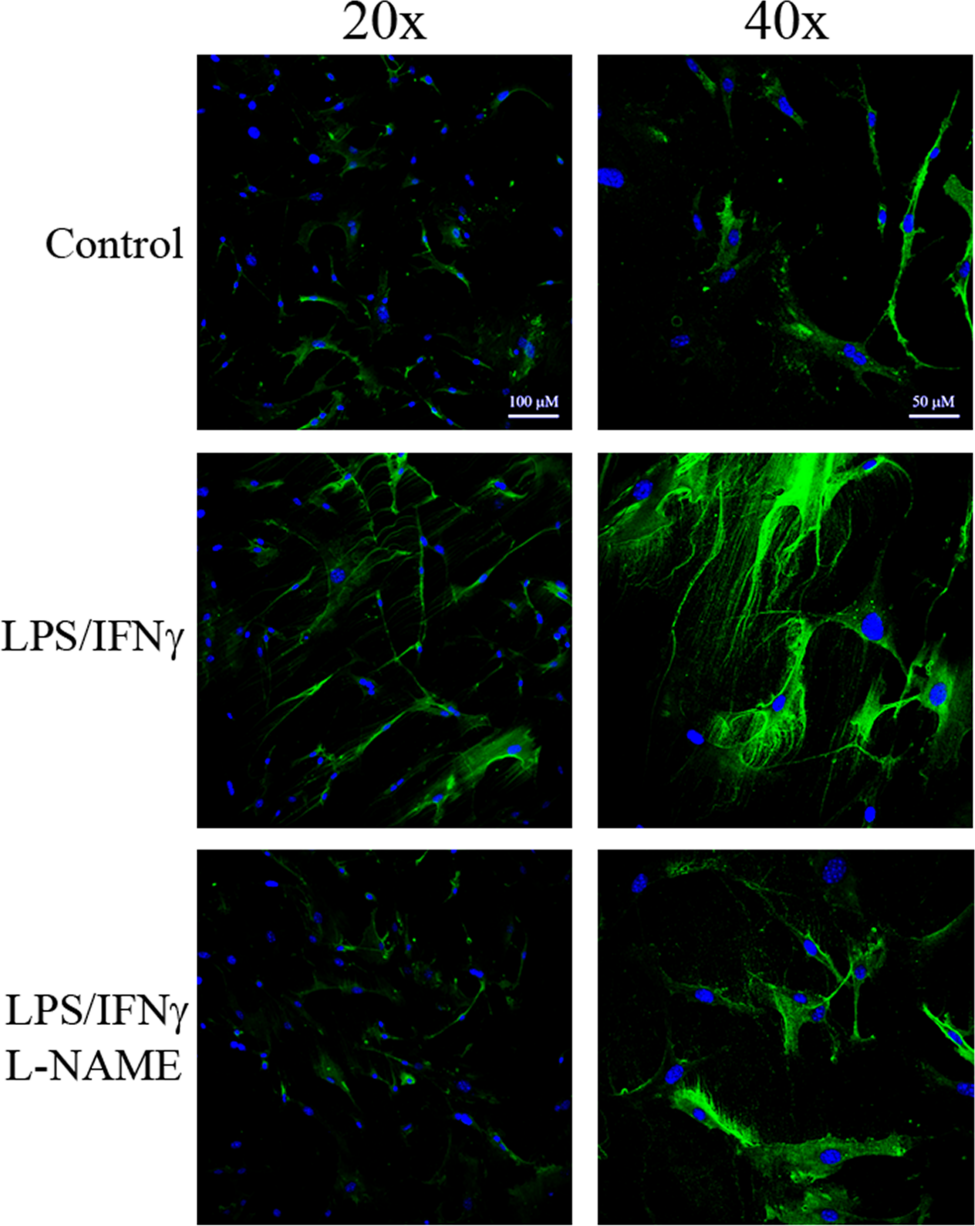
SMCs co-cultured with induced RAW cells produce HA cables. RAW cells were grown on trans-well inserts and treated with 1 ng/ml LPS and 10 U/ml IFN-γ for 6–8 h to induce iNOS expression. Some cultures were also treated with L-NAME. The inserts were rinsed and transferred to plates containing coverslips with confluent SMCs. The RAW cells and SMCs were grown in co-culture for 13 h and the SMCs were then fixed and stained for HA (green) and nuclei (blue). Original magnification 20x (left panels) or 40x (right panels). Images shown are representative of three separate experiments.

While a variety of chemical NO donors promote the deposition of HA cable structures by SMCs, we wanted to determine if NO provided by a cellular source was sufficient to induce HA production. For these studies SMCs were cocultured with a murine macrophage cell line (RAW cells). RAW cells were pre-treated with 1 ng/ml LPS and 10 U/ml interferon gamma (IFN-γ) to promote inducible nitric oxide synthase (iNOS) expression and NO production[65]. Some cultures were also treated with 3 mM L-NAME to inhibit NO production from iNOS. The NO-producing RAW cells were grown in co-culture with SMCs for 13 hours and then the SMCs were harvested and stained for HA. The SMCs exposed to LPS/IFN-γ-treated RAW cells deposit numerous, insoluble HA cable structures (Fig 10). Few of these HA structures are observed in the SMC exposed to untreated RAW cells (Fig 10). Treatment of the RAW cells with L-NAME before and during co-culture reduced the production of HA cables, consistent with a role for NO in promoting HA cable formation (Fig 10).

**Fig 11:**
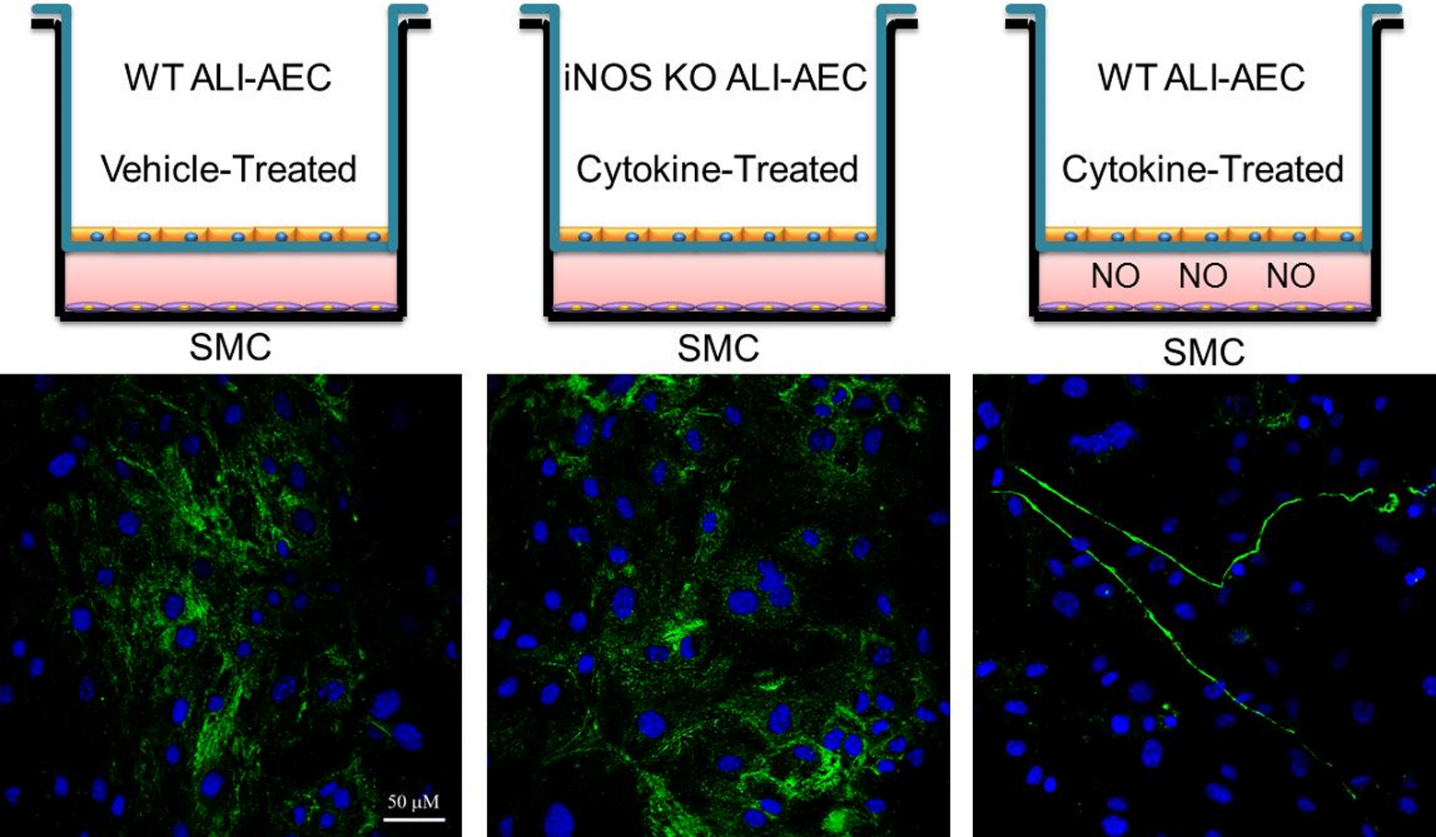
SMCs co-cultured with cytokine-induced airway epithelial cells in an airway organotypic model produce HA cables. Wild type and *iNOS* knockout airway epithelial cells were grown on an air/liquid interface on trans-well inserts and were treated with either vehicle (PBS containing 0.1% BSA) or vehicle containing 10 ng/ml IL-1β, 10 ng/ml TNF-α and 10 ng/ml IFN-γ for 24 hr. The inserts were gently washed and transferred to plates containing SMCs grown on coverslips. The co-cultures were maintained for 23 h and the conditioned medium was collected and the SMCs were stained for HA (green) and nuclei (blue). Original magnification 40x. Experiments were performed three separate times.

Although the induced RAW cells produce NO, other mediators may also be secreted that could potentially affect HA production by SMC. We therefore performed additional co-culture experiments with airway epithelial cells from wild type and *iNOS* knockout mice grown on an air/liquid interface (ALI). The ALI epithelial cells were pre-treated with a cytokine mix known to induce NO production (10 ng/ml IL-1β, 10 ng/ml TNF-α and 10 ng/ml IFN-γ)[61] and were then co-cultured with wild type SMCs. NO concentrations in the co-cultures were 116 μM for wild type, cytokine-treated; 23 μM for wild type, vehicle-treated; and 29 μM for iNOS KO, cytokine-treated samples. SMCs exposed to the cytokine-treated, NO-producing, wild type epithelial cells deposit HA cables while those exposed to the treated iNOS KO epithelial cells do not (Fig 11), consistent with a causal role for NO.

## Discussion

Asthma is a prevalent, chronic inflammatory disorder of the airway. Airway SMCs have been a major therapeutic target in asthma not only because of their roles in airway hyper-responsiveness, but also because of the key roles they play in inflammation and remodeling. Identifying novel cellular and molecular mechanisms involved in airway inflammation and remodeling is needed to better understand this complex disease and for the development of effective treatments.

Hyaluronan accumulates in a variety of inflammatory conditions, including asthma. Hyaluronan is elevated in broncho-alveolar lavage fluid as well as in the airway extracellular matrix of asthmatics. Recently it was shown that circulating HA correlates with airway resistance and may be a marker of asthma control[66] and sputum HA levels may be an additional biomarker[24]. We previously employed a mouse model of ovalbumin-induced asthma to examine HA deposition *in vivo*[67]. Hyaluronan was increased in broncho-alveolar lavage fluid and lungs within 24 hours of challenge and often colocalized with inflammatory cells[67]. Hyaluronan likely has numerous roles in the pathogenesis of asthma including the retention of immune cells. Unfortunately, it is not clear which specific factors may be responsible for the increased deposition of HA.

Nitric oxide is elevated in many allergic asthmatics and is associated with inflammation and worsening asthma[41,42,44] and also identifies a reactive, at risk asthma phenotype[43]. Our *in vitro* results demonstrate that exposure to NO donors or NO produced by stimulated epithelial cells or macrophages leads to enhanced HA deposition by airway SMCs and HA-dependent leukocyte binding. NO is a biomarker of inflammation, particularly eosinophilic inflammation, and plays an active role in the pathogenesis of asthma. Our results, demonstrating increased deposition of HA by SMCs treated with NO, and consequent leukocyte binding, provides further evidence of a role for NO in the pathogenesis of asthma, and importantly points to NO in the abnormal extracellular matrix that is fundamental for pathologic asthmatic airway remodeling. The SMCs responded rapidly to NO by depositing an HA-rich extracellular matrix capable of binding leukocytes. After 30 min of treatment what appear to be emerging cables are evident and by one hour the SMC cultures contained numerous HA cables. This observation, combined with the deposition of HA in the absence of protein synthesis, and no change in HAS2 protein levels, suggests a role for the modification and activation of one or more of the hyaluronan synthase enzymes rather than de novo synthesis of more enzyme. NO may be directly modifying one of the enzymes, or signaling events induced by NO may lead to other modifications such as phosphorylation.

Three mammalian enzymes synthesize hyaluronan; HAS1, HAS2 and HAS3[68,69]. The roles of the various enzymes during inflammation have not been delineated, but a recent genome wide association study has shown *HAS2* is a susceptibility gene for asthma in a Japanese population[14]. Nitric oxide treatment of airway SMCs from the *HAS 1*,*3* double knockout results in the production of abundant leukocyte-adhesive HA cables, demonstrating that HAS2 is sufficient to mediate the effects of NO. HAS2 protein levels are unchanged by exposure to NO consistent with an activation of HAS2 rather than an up regulation of its expression. SMCs deficient in HAS2 made substantially fewer and shorter HA cables in response to NO than control SMCs. The ability of HAS2 to generate leukocyte-binding HA cables in response to NO may contribute to its association with asthma.

Ovalbumin-challenged transgenic mice overexpressing HAS-2 in SMCs and myofibroblasts displayed increased fibrosis, but reduced airway hyperreactivity, demonstrating the complex and numerous effects of HA on lung pathology[15]. It has been noted that hyaluronan size and structure determine its function[70,71]. Low molecular weight HA elicits very different effects than high molecular weight HA. Hyaluronan in the form of cables *in vitro* may have different functions than HA in other structural forms and *in vivo*[70,72]. Numerous factors can increase HA production, but few factors have been identified that promote the deposition of hyaluronan in the form of cables (described as a pathological form, pro-inflammatory form). The HA produced by overexpressing the HAS2 enzyme may be in a very different size and form than the HA produced as a consequence of exposure to nitric oxide. High molecular weight HA has been shown to be anti-inflammatory, consistent with the results described by Walker et al. in their mouse model. Hyaluronan produced by overexpressed HAS2 may not be associated with other matrix components as it can be when present in the form of cable structures[29,31,73] since other components are not being overexpressed. The lack of interaction with other components likely imparts the HA with effects distinct from the effects of the pathological HA produced by NO exposure.

Soluble guanylate cyclase mediates many of the effects of NO, but we found that activation of sGC by BAY compounds, stabilization of cGMP by inhibiting phosphodiesterase-5, or direct addition of a stable analogue of cGMP did not induce HA deposition by SMCs, but addition of tunicamycin to produce ER stress could increase NO-mediated HA cable production and leukocyte binding. Likewise, ODQ, a potent irreversible inhibitor of sGC, did not prevent HA cable formation by SMCs treated with NOC-18. Taken together these results are consistent with an sGC-independent mechanism of NO-induced HA deposition. Consistent with these findings, GSNO, which provides NO by transfer reactions rather than by releasing NO, also leads to the production of HA cables by airway SMCs.

We previously demonstrated that compounds that promote the unfolded protein response (ER stress) in SMCs lead to the deposition of HA-containing cable structures that are capable of binding leukocytes[29,74]. When SMCs with ER stress are also exposed to NO, there is even more HA deposition and leukocyte adhesion than in either the stressed cells alone or those treated with only NO. The *ORMDL3* gene, which encodes a transmembrane protein of the ER, has been shown to be associated with asthma in several studies. This protein facilitates the unfolded protein response that is induced in cells with ER stress. Asthmatics with the *ORMDL3* mutation may have continuously low levels of ER stress and may be particularly sensitive to the effects of NO, resulting in even higher HA production than other asthmatics. Interestingly, the *ORMDL3* gene has been associated with severe asthma[75,76].

Our results demonstrate that NO regulates HA deposition and interestingly, HA has also been shown to regulate iNOS expression and NO production in a variety of cell types and biological systems. The response to HA is dependent on its size, with fragments typically upregulating iNOS, and high molecular weight HA downregulating it[77–83]. Perhaps the deposition of HA into cable structures by NO is a means to prevent or postpone an escalating cycle of increasing HA fragments causing further increases in NO that again affects HA.

Recruitment and retention of inflammatory cells into the airway are key elements in the pathogenesis of asthma. While numerous studies have addressed the recruitment of inflammatory cells into sites of inflammation, little is known regarding how these cells are retained in the tissue. Our data show that NO-promoted HA cables may serve as anchoring sites for migrating leukocytes. Increased HA deposition by NO may initially be advantageous, and could perhaps serve in a protective role, but when produced in excess over long periods of time could become maladaptive and pathologic. The structural form of HA is likely another important factor in determining if excess HA is advantageous or pathological since only the cable-like form of HA binds leukocytes. Perhaps excess non-cable HA is beneficial because it would reduce the tensile strength of the tissue and protect against bronchoconstriction, while excess cable HA is pathologic because of its leukocyte binding properties. Understanding the precise mechanisms that regulate the various structural and functional forms of HA production could be therapeutically useful in order to modulate airway HA concentrations to obtain the optimal concentrations and structural forms.

In conclusion, NO delivered to SMCs by a variety of sources, including cellular sources, results in the production of leukocyte-adhesive HA structures. Our findings take seemingly disparate risk factors and features of asthma, namely NO, HA (HAS2), ER stress (ORMDL3) and SMCs and demonstrate their interactions.
